# The Key Metabolites in Rice Quality Formation of Conventional *japonica* Varieties

**DOI:** 10.3390/cimb45020064

**Published:** 2023-01-20

**Authors:** Qiangqiang Xiong, Changhui Sun, Runnan Wang, Ruizhi Wang, Xiaoyu Wang, Yu Zhang, Jinyan Zhu

**Affiliations:** 1 Jiangsu Key Laboratory of Crop Genetics and Physiology/Jiangsu Key Laboratory of Crop Cultivation and Physiology, Agricultural College of Yangzhou University, Yangzhou 225009, China; 2 Jiangsu Co-Innovation Center for Modern Production Technology of Grain Crops, Yangzhou University, Yangzhou 225009, China

**Keywords:** conventional *japonica* rice, metabolomics, rice quality, carbohydrates, amino acid, metabolic pathway

## Abstract

To understand differences in the quality of different conventional *japonica* rice varieties and variations in metabolites related to rice quality, the quality of three conventional *japonica* varieties was determined, and the metabolites of the milled rice were investigated using nontargeted metabolomics technology. The results showed that the taste value (TV) of Yangda 4Hao (YD4) was significantly higher than that of Yangda 3Hao (YD3) and Huaidao 5Hao (HD5). The protein content (PC) of HD5 was significantly higher than that of YD3 and YD4. PC was significantly negatively correlated with TV. Ninety-one differential metabolites (59 increased and 32 decreased) were identified between YD3 and HD5. A total of 144 differential metabolites (96 upregulated and 48 downregulated) were identified between YD4 and HD5. A total of 114 differential metabolites (40 increased and 74 decreased) were identified between YD3 and YD4. The metabolites with a high correlation to rice quality were mostly involved in the amino acid metabolism pathway. Amino acid metabolites play an important role in the formation of rice quality. The key metabolites in the synthesis and regulation of metabolic pathways are sucrose, levan, and amylose, which are carbohydrates, and L-glutamine, L-aspartic acid, and L-asparagine, which are amino acid metabolites. It can be seen from this study that the metabolites of sucrose, levan, amylose, L-glutamine, L-aspartic acid, and L-asparagine may be the key metabolites in the quality formation of high-quality rice varieties.

## 1. Introduction

For half a century, to solve the problem of food and clothing, the main goal of rice breeding and cultivation has been to increase yield [1]. With the improvement in living standards, consumers pay increasing attention to the quality of rice, and the contradiction between the supply and demand of high-quality rice, especially high-quality edible rice, has become progressively more prominent [2,3]. Under the premise of ensuring food security, “focusing on the development of a high-quality rice industry” has become an inevitable choice for the long-term development of the rice industry [4]. The main components of rice, such as amylose, protein, lipid, vitamin, and amino acid metabolites, are closely related to rice quality [5]. Therefore, studying changes in metabolites related to rice quality among different varieties is helpful for understanding the metabolic mechanisms that drive the improvement of rice quality. At present, many studies focus on metabolic gene regulation mechanisms. The oil content of high-quality rice varieties is especially important to the taste of rice [6,7]. Previous studies used GC-MS to identify the composition and content of fatty acids in 533 varieties of cultivated rice seeds and found that various fatty acid components had extensive variation and significant differences among cultivated rice subgroups [8]. Through genome-wide association analysis, 46 significant loci were identified, of which 16 were repeatedly detected in 3 RIL populations (recombinant inbred lines), and consequently, *OsPAL6*, *OsLIN6*, *OsMYR2*, and *OsFAE6* were cloned, which significantly affected the oil content and composition of the new genes [8]. Our team previously analysed differences in processing quality, amylose content, and taste value under different nitrogen fertilizer and planting density treatments and the relationships between these factors and metabolites. The difference in lipids may be the key to the change in taste value under different nitrogen fertilizer and density treatments [4]. Further use of metabolomics technology to explore rice quality-related metabolites in different rice varieties is of great significance for breeding new high-quality rice varieties [9].

Nontargeted metabolomics is a comprehensive and systematic analysis of metabolites in biological systems to understand qualitative and quantitative variations in metabolites through the acquisition of large amounts of data and processing to identify the roles of specific metabolites in driving product quality [10]. As a model plant, rice has been extensively studied at the genome, transcriptome, and proteome levels. Metabolites are end products located downstream of gene and protein regulation, but their composition and content directly affect phenotype [11]. The combination of multivariate statistics and liquid chromatography/mass spectrometry can more intuitively reveal changes in metabolites during rice growth [12]. Specific types and levels of metabolites are related to the nutrient composition of crops [13,14]. In this study, three conventional *japonica* rice varieties were used as experimental subjects to identify metabolites in milled rice using nontargeted metabolomic technology and to explore relationships between types of metabolites and the development of rice quality among different varieties. This study provides basic metabolic data for breeding high-quality rice varieties.

## 2. Materials and Methods

### 2.1. Experimental Materials and Planting Management

In this study, three conventional *japonica* rice varieties grown in Jiangsu Province were used as experimental materials, namely, Yangda 3Hao (YD3), Yangda 4Hao (YD4), and Huaidao 5Hao (HD5). The amylose content of the three rice varieties was more than 14%, suggesting they were conventional *japonica* rice varieties rather than semi waxy *japonica* rice varieties. HD5 is often used as the control for rice variety certification in Jiangsu Province. The three rice varieties were planted at the Shatou base of Yangzhou University. Rice seedlings were raised with blanket seedlings, and the seedlings were transplanted for 25 d. Four seedlings were planted in each hole, and three replicates were set for each variety. In this experiment, three rice varieties were planted and field managed in a unified way, and the same control methods were used for diseases, pests, and weeds. Pests and weeds were controlled using conventional field management.

### 2.2. Determination of Rice Quality Traits

After the rice was mature, it was harvested, and three biological replicates were taken for each variety. Rice quality indicators were determined after the grains were held in a naturally dry environment for three months, during which the physicochemical properties of the rice grains were stable. The brown rice rate (BR), milled rice rate (MR), head milled rice rate (HMR), chalkiness rate (CR), chalkiness degree (CD), gel consistency (GC), protein content (PC), and amylose content (AC) were determined according to the national standard of the People’s Republic of China (GB/T17891-2017).

The percentage of brown rice is the percentage by weight of the clean rice in the rice sample after the husk is removed by the husker. The milled rice rate refers to the percentage by weight of brown rice or rice grains in the rice samples after being milled and processed by a rice mill, with the bran layer and embryo removed. The head rice rate is the percentage by weight of the milled rice grains that are intact and whose grain length reaches more than 4/5 of the average length of the whole rice grains in the rice sample.

One hundred milligrams of milled rice flour were accurately weighed and placed into a test tube. 0.2 mL of 95% ethanol thymol blue solution was added, and the test tube was gently shaken to make the rice flour fully dispersed without precipitation and agglomeration. Then, 2.0 mL of a 0.2 mol L^−1^ KOH solution was added, and the test tube was gently shaken. The test tube was placed into a boiling water bath, and the mouth of the test tube was covered with a glass ball to prevent steam from escaping during heating. The test tube was heated for 8 min and then placed into an ice water bath for 20 min. After removing the test tube from the ice bath, it was placed flat on a horizontal table, the length of the rice glue was observed, and the gel consistency was measured.

A total of 0.0500 g of rice flour was weighed and placed in a 50 mL digestive tube. Then, 0.5 mL of 95% ethanol was added, and the volumetric flask was gently shaken to make the sample fully wet and dispersed. Next, 4.5 mL of a 1.00 mol L^−1^ sodium hydroxide solution was added, boiled for 10 min, removed, and cooled to room temperature; the volume was adjusted with distilled water. The sample solution (2.5 mL) was removed and added to a 50 mL digestion tube with 0.5 mL of 1.00 mol L^−1^ acetic acid solution and 0.75 mL iodine solution. The tube was shaken, and the volume was adjusted with distilled water. Samples were allowed to stand for 20 min. Additionally, 2.5 mL of a 0.09 mol L^−1^ sodium hydroxide solution was used to prepare a blank solution. The blank solution was used to adjust the zero point of the spectrophotometer at a wavelength of 620 nm, and the absorbance value of the sample was measured. Then, 0.0500 g of each standard sample was weighed and measured with the sample to be tested. The standard curve was drawn with amylose as the ordinate and the corresponding absorbance value as the abscissa.

The appearance (AP), hardness (HA), viscosity (VI), and balance degree (BD) of the rice were measured by a rice taste metre (STA 1A, Satake, Japan), and the comprehensive scores were calculated to yield the taste value (TV). First, 30 g of milled rice was added to a stainless steel tank, water was added, and the tank was soaked for 30 min. Then, the stainless steel tank was covered with filter paper and sealed with a rubber band. The sample was placed in a cooking pot and cooked for 30 min. After 30 min, the rice was braised in a cooking pot for 10 min. The stainless steel tank was removed, the filter paper was removed, and the rice around the stainless steel tank was gently turned with an iron spoon to prevent the rice from sticking to the tank wall. Then, the filter paper was covered again, and the tank was sealed with a rubber band and placed into the rice taste meter to measure the taste value.

### 2.3. Metabolite Extraction and LC-MS/MS Analysis

The samples of head milled rice (50 mg) were accurately weighed, after which the metabolites were extracted and preprocessed according to the method of Xiong et al. [15]. The metabolites were extracted using a 400 µL methanol:water (4:1, *v*/*v*) solution with 0.02 mg/mL L-2-chlorophenylalanin as an internal standard. The mixture was allowed to settle at −10 °C and treated with a high-throughput tissue crusher, Wonbio-96c (Shanghai Wanbo Biotechnology Co., Ltd., Shanghai, China), at 50 Hz for 6 min, followed by ultrasound at 40 kHz for 30 min at 5 °C. The samples were placed at −20 °C for 30 min to precipitate proteins. After centrifugation at 13,000× *g* at 4 °C for 15 min, the supernatant was carefully transferred to sample vials for LC-MS/MS analysis. The mobile phases consisted of 0.1% formic acid in water:acetonitrile (95:5, *v*/*v*) (solvent A) and 0.1% formic acid in acetonitrile:isopropanol:water (47.5:47.5:5, *v*/*v*) (solvent B). The solvent gradient changed according to the following conditions: from 0 to 3.5 min, 0% B to 24.5% B (0.4 mL min^−1^); from 3.5 to 5 min, 24.5% B to 65% B (0.4 mL min^−1^); from 5 to 5.5 min, 65% B to 100% B (0.4 mL min^−1^); from 5.5 to 7.4 min, 100% B to 100% B (0.4 mL min^−1^ to 0.6 mL min^−1^); from 7.4 to 7.6 min, 100% B to 51.5% B (0.6 mL min^−1^); from 7.6 to 7.8 min, 51.5% B to 0% B (0.6 mL min^−1^ to 0.5 mL min^−1^); from 7.8 to 9 min, 0% B to 0% B (0.5 mL min^−1^ to 0.4 mL min^−1^);from 9 to 10 min, 0% B to 0% B (0.4 mL min^−1^) for equilibrating the systems. The sample injection volume was 2 µL and the flow rate was set to 0.4 mL min^−1^. The column temperature was maintained at 40 °C. During the period of analysis, all these samples were stored at 4 °C. Mass spectrometric data were collected using a Thermo UHPLC-Q Exactive HF-X mass spectrometer (Thermo Fisher Scientific, Waltham, MA, USA) equipped with an electrospray ionization (ESI) source (Agilent Technologies Inc., Santa Clara, CA, USA) operating in either positive or negative ion mode. The optimal conditions were set as follows: heater temperature, 425 °C; capillary temperature, 325 °C; sheath gas flow rate, 325 arb; aux gas flow rate, 13 arb; ion-spray voltage floating (ISVF), −3500 V in negative mode and 3500 V in positive mode; and normalized collision energy, with 20-40-60 V rolling for MS/MS. The full MS resolution was 60,000, and the MS/MS resolution was 7500. Data acquisition was performed in data-dependent acquisition (DDA) mode. The detection was performed over a mass range of 70–1050 *m*/*z*.

After UPLC-MS analyses, the raw data were imported into Progenesis QI 2.3 (Nonlinear Dynamics, Waters, Milford, MA, USA) for peak detection and alignment. The preprocessing results generated a data matrix that consisted of the retention time (RT), mass-to-charge ratio (*m*/*z*) values, and peak intensity. Metabolic features detected in at least 80% of any set of samples were retained. After filtering, minimum metabolite values were imputed for specific samples in which the metabolite levels fell below the lower limit of quantitation, and each metabolic feature was normalized by the sum. Following normalization procedures and imputation, statistical analysis was performed on log-transformed data to identify significant differences in metabolite levels between comparable groups. The mass spectra of these metabolic features were identified by using accurate mass spectrometry, MS/MS fragment spectra, and isotope ratio differences by searching reliable biochemical databases such as the Human Metabolome Database (HMDB) (http://www.hmdb.ca/, accessed on 1 July 2022)) and Metlin Database (https://metlin.scripps.edu/, accessed on 1 July 2022). The mass tolerance between the measured *m*/*z* values and the exact mass of the components of interest was ±10 ppm. For metabolites with MS/MS confirmation, only those with MS/MS fragment scores above 30 were considered confidently identified. Otherwise, metabolites had only tentative assignments.

### 2.4. Metabolite Statistical Analysis

A multivariate statistical analysis was performed using the ropls (Version 1.6.2, http://bioconductor.org/packages/release/bioc/html/ropls.html, accessed on 1 July 2022) R package from Bioconductor on the Majorbio Cloud Platform (https://cloud.majorbio.com, accessed on 1 July 2022) [16]. Principal component analysis (PCA) is a technology for simplifying data analysis. This method can effectively find the most “major” elements and structures in data, remove noise and redundancy, and reduce the dimensions of original complex data [17]. All of the metabolite variables were scaled to unit variances prior to conducting the PCA. Partial least squares discrimination analysis (PLS-DA) is a supervised discriminant analysis statistical method [18]. In this method, PLS-DA was used to establish the relationship model between the expression of metabolites and sample categories so as to realize the prediction of sample categories. All of the metabolite variables were scaled to Pareto scaling prior to conducting the OPLS-DA. The model validity was evaluated using the model parameters R2 and Q2, which provide information for the interpretability and predictability, respectively, of the model and avoid the risk of overfitting. The variable importance in the projection (VIP) was calculated in the OPLS-DA model. *p*-values were estimated with a paired Student’s *t* test on single-dimensional statistical analysis. Differential metabolites (DMs) were screened based on variable importance for projection (VIP) values greater than 1 and *p* values less than 0.05. Multivariate statistical analysis was performed using the ropls R package (Version 1.6.2, http://bioconductor.org/packages/release/bioc/html/ropls.html, accessed on 1 July 2022) from Bioconductor on the Majorbio Cloud Platform (https://cloud.majorbio.com, accessed on 1 July 2022). The DMs were mapped onto KEGG pathways based on a database search (KEGG, http://www.genome.jp/kegg/, accessed on 1 July 2022).

### 2.5. Data Analysis

WPS 2021 software (Beijing Kingsoft Office Software, Inc., Beijing, China) was used to organize and calculate the average value of rice quality data, SPSS 18.0 software (SPSS, Chicago, UL, USA) was used to perform variance analysis of rice quality, and Adobe Illustrator CS6 software (Adobe Systems Incorporated, San Jose, CA, USA) was used to combine each graph.

## 3. Results

### 3.1. Analysis of Rice Quality Traits

There are some differences in rice quality traits among the three conventional *japonica* rice varieties (Table 1). BR was the highest in HD5, in which the BR was 2.8% and 1.29% higher than that in YD3 and YD4, respectively, and the difference was significant. Compared with YD3 and YD4, the HMR of HD5 increased by 3.13% and 18.69%, respectively. MR was highest in YD4 at 5.23% and 1.75% higher than that in YD3 and HD5, respectively, and the difference was significant. Compared with HD5, the CR and CD of YD3 were decreased by 51.24% and 56.4%, respectively, and the CR and CD of YD4 were decreased by 51.88% and 65.97%, respectively. Compared with YD3 and YD4, the GC of HD5 decreased by 0.41% and 6.63%, respectively. The AP, VI, BD, and TV of YD4 were significantly higher than those of YD3 and HD5. The TV of YD4 was 8.5% and 9.37% higher than that of YD3 and HD5, respectively. Compared with YD3 and YD4, the AC of HD5 was increased by 2.65% and 4.94%, respectively. The PC of HD5 was 8.1% and 13.98% higher than that of YD3 and YD4, respectively, and the difference was significant. A correlation analysis was conducted on the rice quality trait data. CR was significantly positively correlated with CD. HA was significantly negatively correlated with AP, VI, BD, and TV. TV was significantly positively correlated with AP, VI, and BD. PC was significantly negatively correlated with TV (Table S1).

### 3.2. Multivariate Statistical Analysis

**Figure 1:**
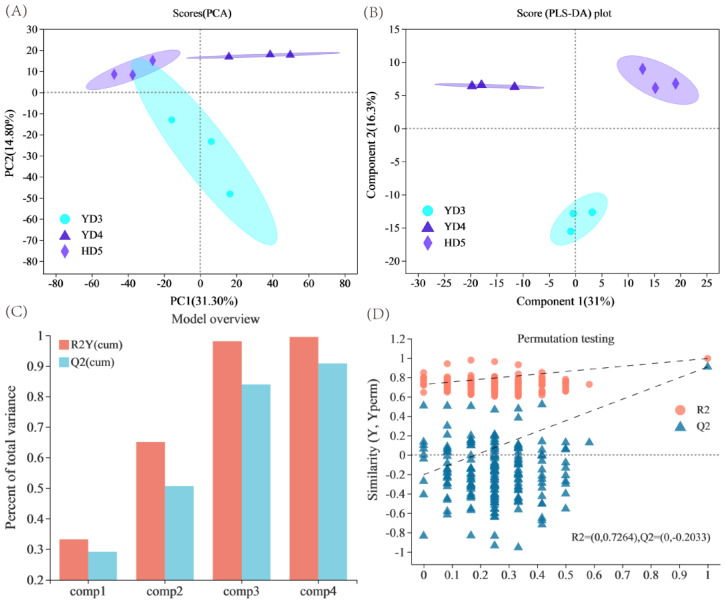
Multivariate statistical score and permutation testing among the four rice varieties. (**A**) PCA. (**B**) PLS-DA. (**C**) Overview of the PLS-DA model. (**D**) PLS-DA permutation testing.

Rice grains have abundant metabolites, but there are few studies comparing metabolite differences among rice varieties with different tastes. The three conventional japonica varieties had a total of 215 identical metabolites. On the PCA score plot, the two principal components (PC1) and PC2 accounted for 31.3% and 14.8%, respectively (Figure 1A). The PCA results showed that there were differences in metabolites among the three conventional *japonica* varieties. To obtain information on the metabolites responsible for these significant differences, supervised multidimensional statistical methods were used to analyse the samples. Partial least squares discriminant analysis (PLS-DA) demonstrated that component 1 and component 2 could explain 31% and 16.3% of the variation, respectively (Figure 1B). The cumulative interpretation rate of the PLS-DA model was 0.874 for R2X (cum), 0.995 for R2Y (cum), and 0.908 for Q2 (cum). The analysis showed that the PLS-DA model had better explanatory power and a stronger ability to predict data. All samples are within the confidence interval. Compared with the PCA score, the three groups of samples do not overlap, and the separation effect is clearer. After 200 random permutation tests, with the decrease in permutation retention, R2 and Q2 decreased, and the regression line showed an upward trend, indicating that the permutation test passed the test and that the model did not have overfitting.

### 3.3. Metabolic Profiling

**Figure 2:**
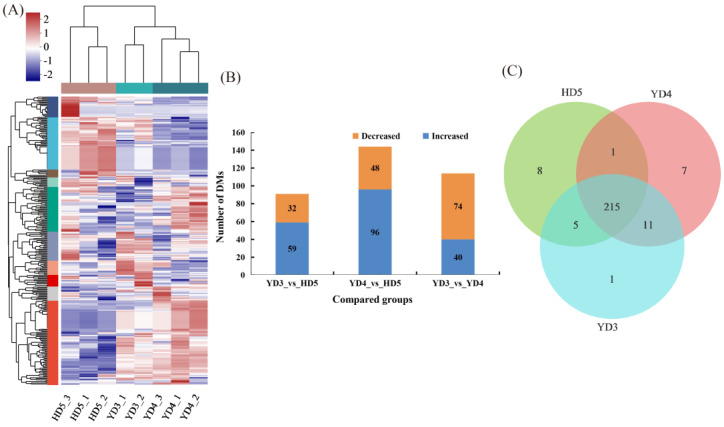
Hierarchical clustering heatmap and metabolite analysis. (**A**) Hierarchical clustering heatmap among three *japonica* rice samples; (**B**) pairwise comparison of differential metabolites; and (**C**) metabolite Venn distribution map.

The heatmap of cluster analysis can be used to understand the accumulation of different metabolites among different varieties as a whole. The metabolite of the YD3_3 sample deviated greatly, so the analysis was conducted after removing the YD3_3 sample. There were significant differences in the accumulation of metabolites among different varieties, and the biological repetitions of each group were highly similar (Figure 2A). Ninety-one DMs (59 increased and 32 decreased) were identified in the YD3 and HD5 comparison groups (Figure 2B; Table S2). Glycerophospholipids accounted for 14.06%, prenol lipids accounted for 7.81%, glycerolipids accounted for 6.25%, phenols accounted for 1.56%, and flavonoids accounted for 1.56% (Figure S1A). A total of 144 DMs (96 increased and 48 decreased) were identified in the YD4 and HD5 comparison groups (Figure 2B; Table S3). Prenol lipids accounted for 6.98%, glycerophospholipids accounted for 5.81%, flavonoids accounted for 2.33%, and phenols accounted for 1.16% (Figure S1B). A total of 144 differential metabolites (96 increased and 48 decreased) were identified in the YD3 and YD4 comparison groups (Figure 2B; Table S4). Glycerophospholipids accounted for 11.69%, prenol lipids accounted for 7.79%, and flavonoids accounted for 1.3% (Figure S1C). Sample correlation analysis indicated that the sample correlation coefficient was high, suggesting that the sample repeatability was good (Figure S2A). Each variety also had unique metabolites (Figure S2B–D).

### 3.4. KEGG Pathway

The DMs of the YD3 and HD5 comparative groups participated in alanine, aspartate, and glutamate metabolism, alpha-linolenic acid metabolism, and tryptophan metabolism (Table 2). The DMs of the YD4 and HD5 comparative groups participated in pantothenate and CoA biosynthesis; alanine, aspartate, and glutamate metabolism; starch and sucrose metabolism; tryptophan metabolism; and glycerophospholipid metabolism (Table 2). The DMs of the YD4 and HD5 comparative groups participated in pantothenate and CoA biosynthesis, alpha-linolenic acid metabolism, starch and sucrose metabolism, and beta-alanine metabolism (Table 2).

### 3.5. Correlation Analysis between Rice Quality Traits and Metabolites

**Figure 3:**
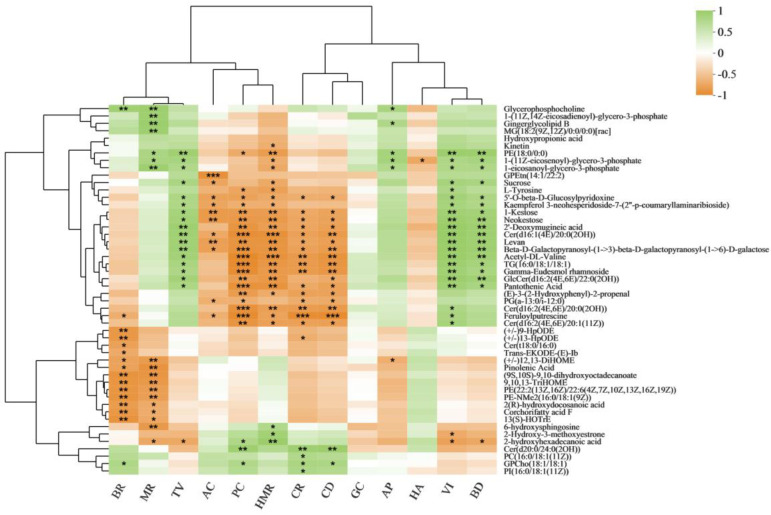
Correlation analysis of rice quality traits and DMs. * *p* < 0.05, ** *p* < 0.01, *** *p* < 0.01.

**Figure 4:**
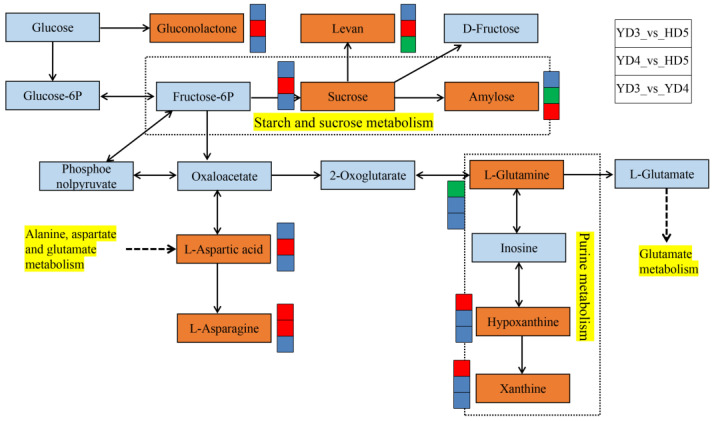
Overview of key metabolites in the metabolic pathways of three conventional *japonica* rice varieties. The key metabolites are shown in orange rectangles. Red squares indicate significant upregulation of the metabolite content; green squares indicate significant downregulation of the metabolite content; blue squares indicate no significant difference in the metabolite content. Yellow represents the metabolic pathways.

We analysed correlations between rice quality traits and metabolites (Figure 3). Glycerophosphocholine was significantly positively correlated with BR, MR, and AP. Sucrose was significantly positively correlated with VI, BD, and TV. Sucrose was significantly negatively correlated with AC and HMR. L-Tyrosine was significantly negatively correlated with PC. Levan was significantly negatively correlated with HMR, CR, CD, AC, and PC. Levan was significantly positively correlated with VI, BD, and TV. 5′-O-beta-D-Glucosylpyridoxine, 1-kestose, neokestose, acetyl-DL-valine, gamma-eudesmol rhamnoside, and pantothenic acid were significantly positively correlated with TV. 5′-O-beta-D-Glucosylpyridoxine, 1-kestose, neokestose, and feruloylputrescine were significantly negatively correlated with AC and PC. Additionally, we mapped metabolic pathway regulation for key metabolites (Figure 4). Glycerophosphocholine is a lipid substance. Sucrose, levan, and amylose are carbohydrates. L-glutamine, L-aspartic acid, and L-asparagine are amino acids (Table S5).

## 4. Discussion

Rice quality includes factors related to processing, appearance, nutrition, and eating quality, among which appearance quality is a crucial component of rice quality while eating quality is the core indicator of high-quality rice [1]. Typically, the indicators used to measure the quality of rice processing are the brown rice rate, milled rice rate, and head milled rice rate. The most important processing quality is the head milled rice rate [19]. In this study, HD5 had the highest BR and HMR. The BR of HD5 was significantly higher than that of YD3 and YD4 (Table 1). This also shows that the processing quality of HD5 varieties is better than that of YD3 and YD4. The chalkiness rate and chalkiness are the core indicators used to evaluate the appearance quality of rice. In this study, the CR and CD of HD5 were significantly higher than those of YD3 and YD4 (Table 1). This also shows that the appearance quality of HD5 varieties is lower than that of YD3 and YD4. Among the three *japonica* varieties, although the HD5 variety had better processing quality, the appearance quality was poor. The taste quality of rice is a comprehensive evaluation of sensory indicators such as odour, colour, viscosity, hardness, and elasticity of rice during cooking and eating [20,21]. In this study, the AP, VI, and BD of YD4 were significantly higher than those of YD3 and HD5 (Table 1). Rice hardness is one of the important factors affecting the taste of rice [4]. In this study, HA was significantly negatively correlated with TV (Table S1). The HA of YD4 was lower than that of YD3 and HD5. The TV of YD4 was significantly higher than that of YD3 and HD5 (Table 1). The eating quality of rice is affected by the amylose, protein, and fat contents [7]. The protein content of rice is related to eating quality [22], and in this study, the protein content of rice was negatively correlated with eating quality (Table S1). Rice varieties with a protein content of more than 9% tended to have hard rice and poor palatability. The protein content of Chinese rice is 7% to 9% [23]. Amylose content is an important trait that determines the eating quality of rice, and the level of amylose content is closely related to the softness and stickiness of rice [24]. In this study, the PC of HD5 was significantly higher than that of YD3 and YD4. AC was also the highest, with HD5. This may also be the main reason for the lower taste value of HD5.

Rice grains have abundant metabolites, but there are few studies comparing metabolite differences among rice varieties with different taste qualities [25]. The taste quality and nutritional quality of milled rice are the key indicators used to measure the quality of a rice variety, and they are also good experimental materials to explore the mechanisms involved in the development of rice taste and nutritional quality. However, there are few studies on the nutritional quality of milled rice [4]. In this study, 91 DMs (Figure 2B; Table S2), including amino acids, organic acids, lipids, phenols, and flavonoids, were identified in the YD3 and HD5 comparison groups using untargeted metabolomic techniques (Figure S1A). A total of 144 DMs were identified in the YD4 and HD5 comparison groups (Figure 2B; Table S3), and 114 DMs were identified in the YD3 and YD4 comparison groups (Figure 2B; Table S4). This study identified more metabolites in both quantity and type. Rice grain development involves the process of transporting photosynthetic compounds to grains, which is closely related to yield and quality [26]. During the process of rice quality formation, cell metabolism is active, and different metabolites gradually accumulate in the grain [27]. Our study found that the content of sucrose was significantly increased in the YD4_vs_HD5 comparison group, which is an important component of grain involved in starch and sucrose metabolism, and sucrose was significantly positively correlated with TV. Sucrose was also significantly negatively correlated with AC. However, the amylose content was significantly decreased (Figure 4). This is consistent with the result that the AC content of YD4 was lower than that of HD5 (Table 1). Amino acids are important indicators for evaluating the nutritional value of cereal grains [28]. Among all the retrieved metabolic pathways, the amino acid metabolism pathway accounted for a large proportion, and amino acid and protein contents were closely related to rice quality [29]. In this study, the alanine, aspartate, and glutamate metabolism pathways were significantly enriched (Table 2), and the amino acid metabolites L-glutamine, L-aspartic acid, and L-asparagine played important roles in the synthesis and regulation of metabolic pathways (Figure 4). These differential metabolites all affect rice quality in different ways. Amino acid metabolism can be used as an important direction for the in-depth study of rice quality formation.

## 5. Conclusions

There are specific variations in the quality and taste value of different conventional *japonica* rice varieties. BR and HMR were the highest in HD5. The CR and CD values of YD3 and YD4 were significantly lower than those of HD5. The PC of HD5 was significantly higher than that of YD3 and YD4. PC was significantly negatively correlated with TV. In the three rice varieties, we inferred that an elevated PC content is the primary factor affecting the taste of the rice. Sucrose was significantly positively correlated with TV. In addition, sucrose is also a key metabolite for metabolism pathway synthesis and regulation in high-quality rice varieties. Therefore, the improvement of rice’s taste value can be achieved by changing the protein content and the sucrose metabolite content. Amino acid metabolic pathways account for a large proportion of all metabolic pathways, and the amino acid metabolites L-glutamine, L-aspartic acid, and L-asparagine play important roles in the synthesis and regulation of metabolic pathways. This study concluded that the metabolites of subcrosse, L-glutamine, L-aspartic acid, and L-asparagine played an important role in the formation of high-quality rice.

## Figures and Tables

**Table 1:** Difference analysis of three *japonica* rice quality traits.

Test Strain	BR (%)	MR (%)	HMR (%)	CR	CD	GC	AP	HA	VI	BD	TV	AC (%)	PC (%)
YD3	83.29 c	68.89 c	64.59 a	34.36 b	14.40 b	80.00 a	5.57 b	6.93 a	5.53 b	5.47 b	63.07 b	16.98% a	7.77% b
YD4	84.53 b	72.49 a	56.12 b	33.91 b	11.24 b	85.33 a	6.30 a	6.73 a	6.67 a	6.33 a	68.43 a	16.61% a	7.37% c
HD5	85.62 a	71.24 b	66.61 a	70.47 a	33.03 a	79.67 a	5.77 b	6.90 a	5.27 b	5.37 b	62.57 b	17.43% a	8.40% a

Note: different lowercase letters indicate significant differences at the 0.05 level. BR, brown rice rate; MR, milled rice rate; HMR, head milled rice rate; CR, chalkiness rate; CD, chalkiness degree; GC, gel consistency; PC, protein content; AC, amylose content; AP, appearance; HA, hardness; VI, viscosity; BD, balance degree; and TV, taste value.

**Table 2:** KEGG pathways associated with DMs.

Pathway Description	Pathway ID	Ratio_in_Pop	*p* Value	Metabolites
KEGG pathways between YD3 and HD5				
Alanine, aspartate, and glutamate metabolism	map00250	28/4834	0.0127	C00152; C00064
alpha-Linolenic acid metabolism	map00592	44/4834	0.0024	C01226; C00157; C16316
Tryptophan metabolism	map00380	83/4834	0.0142	C00978; C05635; C02693
KEGG pathways between YD4 and HD5				
Pantothenate and CoA biosynthesis	map00770	30/4834	0.0155	C00049; C00864
Alanine, aspartate, and glutamate metabolism	map00250	28/4834	0.0136	C00049; C00152
Starch and sucrose metabolism	map00500	37/4834	0.0016	C06215; C00089; C00718
Tryptophan metabolism	map00380	83/4834	0.0155	C00978; C05635; C02693
Glycerophospholipid metabolism	map00564	56/4834	0.0495	C00344; C00157
KEGG pathways between YD3 and YD4				
alpha-Linolenic acid metabolism	map00592	44/4834	0.0319	C00157
Starch and sucrose metabolism	map00500	37/4834	0.0231	C06215; C00089; C00718
beta-Alanine metabolism	map00410	32/4834	0.0175	C00049; C00864

Note: pathway description, KEGG pathway name description, pathway ID, KEGG pathway ID, Ratio_in_pop, the proportion of metabolites annotated to the background pathway in the background metabolites, the number of the background metabolic set annotated to the left of the diagonal, and the number of KEGG compound IDs of the background metabolic set annotated to all pathways; *p* < 0.05 as a significant enrichment term; Metabolites, the KEGG compound IDs corresponding to the metabolites in the pathway are annotated in the metabolic set.

## Data Availability

Not applicable.

## Supplementary Materials

The following supporting information can be downloaded at https://www.mdpi.com/article/10.3390/cimb45020064/s1, Figure S1. Statistical map of compounds. The name and percentage of the metabolites in the selected HMDB hierarchy (class) are displayed in the order of high to low, depending on the number of metabolites. (A) YD3_vs_HD5; (B) YD4_vs_HD5; (C) YD3_vs_YD4; Figure S2. Sample repeatability and DM volcano map. Each dot represents a specific metabolite, and the size of the dot represents the VIP value. On the left are differentially downregulated metabolites, and on the right are differentially upregulated metabolites; the further left or right a point is, the more significant the point above. (A) Heatmap of correlations among three japonica rice samples; (B) YD3_vs_HD5; (C) YD4_vs_HD5; and (D) YD3_vs_YD4; Table S1. Correlation analysis of rice quality traits; Table S2. DM identification information between YD3_vs_HD5; Table S3. DM identification information between YD4_vs_HD5; Table S4. DM identification information between YD3_vs_YD4; Table S5. Detailed information on key metabolites associated with rice quality.
