# Supplementary material for: The Key Metabolites in Rice Quality Formation of Conventional japonica Varieties
Source: Curr Issues Mol Biol. 2023 Jan 20;45(2):990–1001. doi: 10.3390/cimb45020064 (PMC9955130; doi:10.3390/cimb45020064)
Supplement: Supplementary file 1 [file cimb-45-00064-s001.zip › Figure S1.pdf]

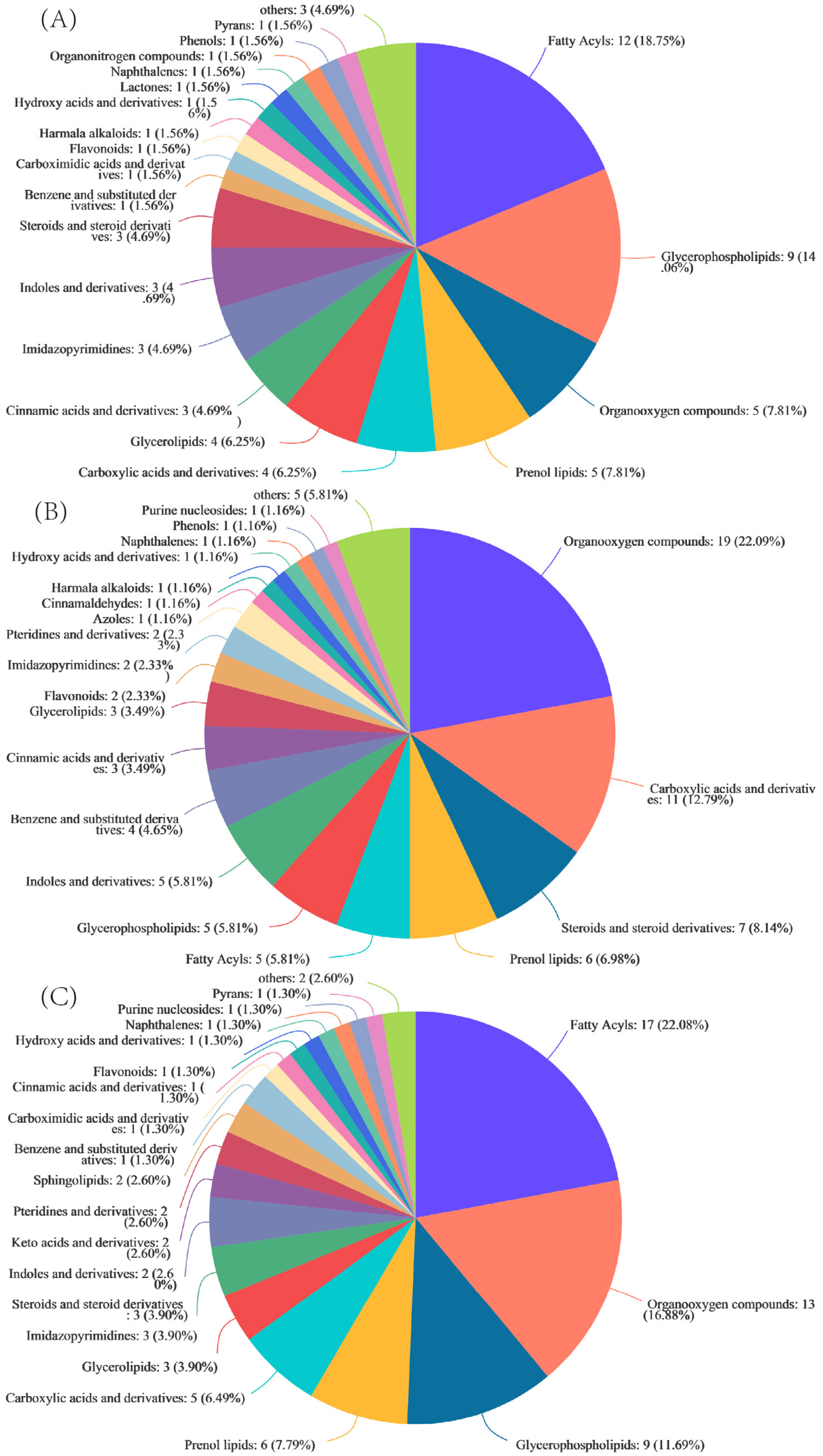

Figure S1. Statistical map of compounds. The name and percentage of the metabolites of the selected HMDB hierarchy (Class) are displayed in the order of high to low, depending on the number of metabolites. (A) YD3_vs_HD5; (B) YD4_vs_HD5; (C) YD3_vs_YD4.
