# Supplementary material for: The Key Metabolites in Rice Quality Formation of Conventional japonica Varieties
Source: Curr Issues Mol Biol. 2023 Jan 20;45(2):990–1001. doi: 10.3390/cimb45020064 (PMC9955130; doi:10.3390/cimb45020064)
Supplement: Supplementary file 1 [file cimb-45-00064-s001.zip › Figure S2.pdf]

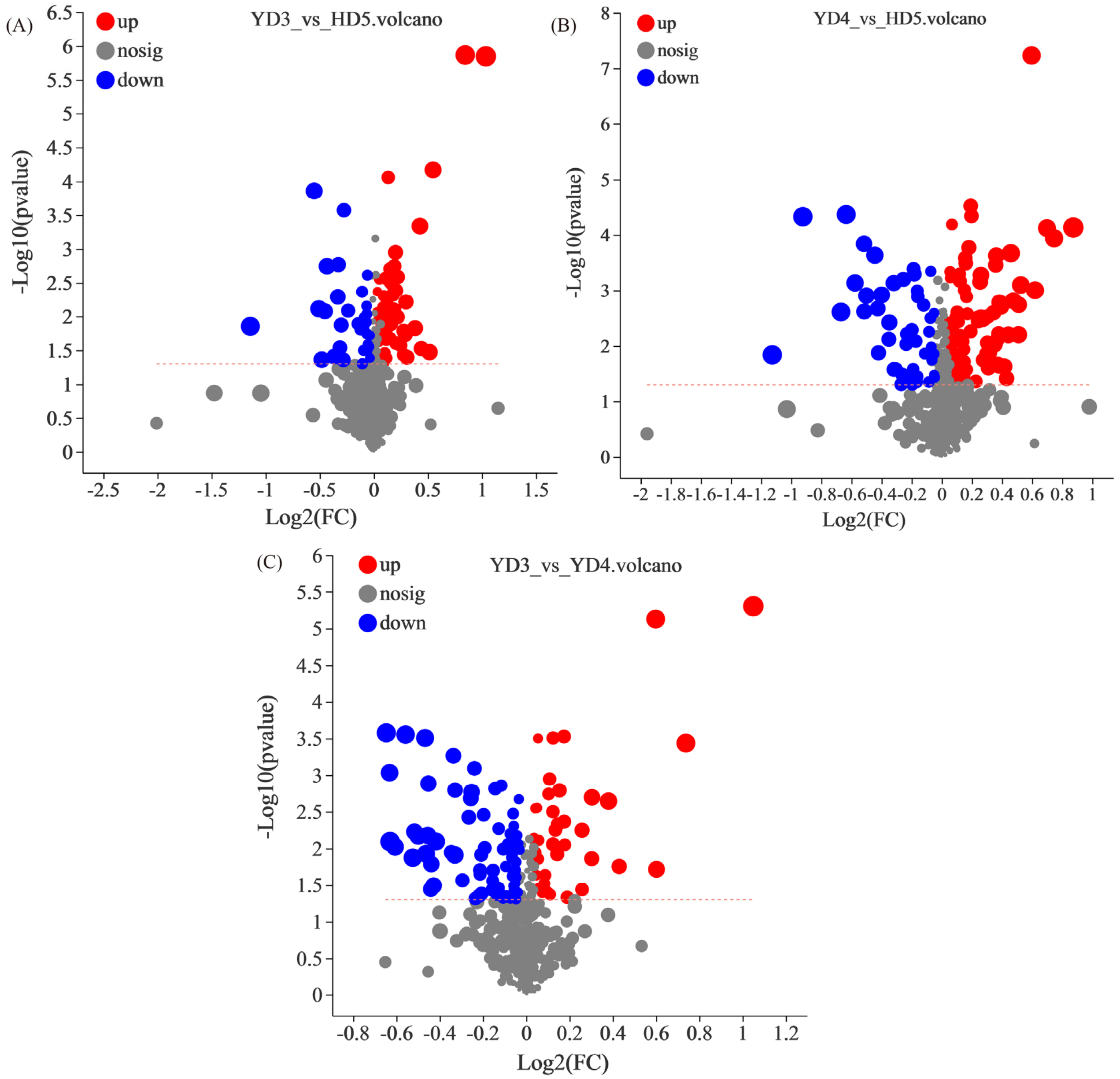

Figure S2. DM volcano map. Each dot represents a specific metabolite, and the size of the dot represents the VIP value. On the left are differentially downregulated metabolites, and on the right are differentially upregulated metabolites; the further left or right a point is, the more significant the point above. (A) YD3_vs_HD5; (B) YD4_vs_HD5; (C) YD3_vs_YD4.
