# Supplementary material for: The Key Metabolites in Rice Quality Formation of Conventional japonica Varieties
Source: Curr Issues Mol Biol. 2023 Jan 20;45(2):990–1001. doi: 10.3390/cimb45020064 (PMC9955130; doi:10.3390/cimb45020064)
Supplement: Supplementary file 1 [file cimb-45-00064-s001.zip › Table S1.pdf]

Table S1. Correlation analysis of rice quality traits.

Character	BR	MR	HMR	CR	CD	GC	AP	HA	VI	BD	TV	AC	PC
BR		0.645	0.149	0.824**	0.744*	0.031	0.276	-0.151	-0.125	-0.038	-0.019	0.354	0.568
MR			-0.566	0.16	0.026	0.444	0.752*	-0.522	0.58	0.621	0.621	0.006	-0.221
HMR				0.648	0.725*	-0.372	-0.691*	0.551	-0.916**	-0.865**	-0.841**	0.606	0.872**
CR					0.990**	-0.244	-0.205	0.206	-0.612	-0.526	-0.499	0.555	0.917**
CD						-0.319	-0.308	0.283	-0.692*	-0.612	-0.585	0.547	0.954**
GC							0.232	-0.115	0.318	0.283	0.263	0.423	-0.377
AP								-0.906**	0.875**	0.935**	0.947**	-0.37	-0.486
HA									-0.761*	-0.846**	-0.865**	0.369	0.403
VI										0.984**	0.979**	-0.556	-0.839**
BD											0.998**	-0.547	-0.756*
TV												-0.526	-0.733*
AC													0.548
PC													

*$P < 0.05$, **$P < 0.01$.
